# Mouse microphthalmia‐associated transcription factor (*Mitf*) mutations affect the structure of the retinal vasculature

**DOI:** 10.1111/aos.15140

**Published:** 2022-03-29

**Authors:** Stefán Broddi Daníelsson, Andrea García‐Llorca, Hallur Reynisson, Thor Eysteinsson

**Affiliations:** ^1^ Department of Physiology, Biomedical Center, Faculty of Medicine University of Iceland Reykjavík Iceland

**Keywords:** arterial diameter, fluorescein angiography, fundus imaging, MITF, mouse, retina, vasculature

## Abstract

**Purpose:**

Mice carrying pathogenic variants in the microphthalmia transcription factor (*Mitf*) gene show structural and functional changes in the retina and retinal pigment epithelium. The purpose of this study was to assess the vascular changes in *Mitf* mice carrying pathogenic variants by determining their retinal vessel diameter.

**Methods:**

Mice examined in this study were: B6‐*Mitf* ^mi‐vga9/+^ (*n* = 6), B6‐*Mitf* ^mi‐enu22(398)^ */Mitf* ^mi‐enu22(398)^ (*n* = 6) and C57BL/6J wild type mice (*n* = 6), all 3 months old. Fundus images were taken with a Micron IV camera after intraperitoneal injection of fluorescein salt. Images were adjusted to enhance contrast and a custom written MATLAB program used to extract the mean vascular diameter at a pre‐defined distance from the optic disc. The number of vessels, mean diameter and mean total diameter were examined.

**Results:**

The mean diameter of retinal veins in *Mitf* ^mi‐enu22(398)^ */Mitf* ^mi‐enu22(398)^ mice was 18.8% larger than in wild type (p = 0.026). No differences in the mean diameter of the retinal arteries were found between the genotypes. *Mitf* ^mi‐enu22(398)^ */Mitf* ^mi‐enu22(398)^ mice have 17.2% more retinal arteries (p = 0.026), and 15.6% more retinal veins (p = 0.041) than wild type. A 24.8% increase was observed in the mean combined arterial diameter in mice with the *Mitf* ^mi‐enu22(398)*/*^ *Mitf* ^mi‐enu22(398)^ compared to wild type mice (p = 0.024). A 38.6% increase was found in the mean combined venular diameter in mice with the *Mitf* ^mi‐enu22(398)^ */Mitf* ^mi‐enu22(398)^ pathogenic variation as compared to wild type (p = 0.004). The mean combined retinal venular diameter in the *Mitf* ^mi‐vga9/+^ mice was 17.8% larger than in wild type (p = 0.03).

**Conclusion:**

An increase in vascularization of the retina in *Mitf* ^mi‐enu22(398)^ */Mitf* ^mi‐enu22(398)^ mice was found, indicating an increased demand for blood flow to the retina.

The retinal vasculature plays a vital role in maintaining the viability of the retina and its normal function. However, the state of the retina, and thus any dysfunction or atrophy, can have profound effects on the diameter of individual vessels (Ma et al. 2012; Kim et al. 2013; Eysteinsson et al. 2014) and the bifurcations of the retinal vasculature as a whole (Ma et al. 2012; de Carlo et al. 2016), indicating a complex interdependence between the retinal cells and the vasculature. Retinal atrophies or degenerations in humans, such as retinitis pigmentosa, are known to lead to vascular rarefaction and narrowing of retinal vessels and elevated oxygen saturation (Eysteinsson et al. 2014; Todorova et al. 2016; Guduru et al. 2018; Lang et al. 2019), reduced retinal and choroidal blood flow (Langham & Kramer 1990; Schmidt et al. 2001; Beutelspacher et al. 2011; Kim et al. 2013; Zhang et al. 2013), and reduction in the number of retinal and choroidal capillaries (Milam et al. 1998; Mullins et al. 2012; Lee et al. 2020; Shen et al. 2020). Mouse models of retinal dysfunction or degeneration show alterations in their retinal vasculature that in many cases are similar to those found in human patients with retinal atrophies (Blanks & Johnson 1986; Richter et al. 1998; Wang et al. 2000; Rakoczy et al. 2010; Liu et al. 2016; Charette et al. 2017).

The pathogenic variations leading to retinal degenerations and alterations in the retinal vasculature that have been examined so far involve genes that in most cases are expressed in photoreceptors (Wang et al. 2000; Wright et al. 2010; Veleri et al. 2015), while a minority are expressed exclusively in the retinal pigment epithelium (RPE) (Fletcher et al. 2014; Veleri et al. 2015; Zhang et al. 2017; Roman et al. 2018). Photoreceptor cells, and possibly their interaction with the RPE, are known to influence retinal vascular degeneration in mouse models of both retinal degeneration as well as diabetes, indicating that photoreceptor loss precedes retinal vascular degeneration in these diseases (Liu et al. 2016). Thus, it is unclear whether variants in genes that in the eye are expressed exclusively in the RPE have any significant effect on the structure of the retinal vasculature. The issue is of importance since it is known that the RPE plays a role in retinal and choroidal vascular development and homeostasis, through release of both pro‐angiogenic factors like the vascular endothelial growth factor (VEGF), fibroblast growth factor (FGF) and platelet‐derived growth factor (PDGF), and anti‐angiogenic factors like the pigment epithelium‐derived factor (PEDF) (Sakagami et al. 2001; Rousseau et al. 2003; Martin et al. 2004; Schlingemann 2004; Saint‐Geniez et al. 2009; Scott et al. 2010; Cabral et al. 2017). The gene encoding the microphthalmia‐associated transcription factor (*Mitf*) is expressed solely in the RPE in the eye (Smith & Hamasaki 1994; Nguyen & Arnheiter 2000; Moller et al. 2004; Arnheiter 2010; Garcia‐Llorca et al. 2019).

The Mitf gene is a member of the Mit‐Tfe subfamily of basic‐helix–loop–helix leucine zipper (BHLH‐Zip) family of transcription factors (Goding & Arnheiter 2019), and has been shown to be involved in regulating the development and function of many organs, including eye, ear, immune system, nervous system, kidney formation, bone and skin (Nguyen & Arnheiter 2000; Bauer et al. 2009; Arnheiter 2010; Lu et al. 2010; Gutknecht et al. 2015; Pillaiyar et al. 2017). In eye development specifically, it has a role in boundary formation between the RPE and the neural retina (Nguyen & Arnheiter 2000; Arnheiter 2010). Mice with loss of function pathogenic variations in *Mitf* show a conversion from RPE to neural retina (Bumsted & Barnstable 2000; Nguyen & Arnheiter 2000; Heavner & Pevny 2012). Human diseases so far associated with pathogenic variations in the Mitf gene include Waardenburg syndrome, Tietz syndrome, COMMAD syndrome, and melanoma (Smith et al. 2000; Takeda et al. 2007; Pingault et al. 2010; Ma et al. 2012; Chantarawong et al. 2014; Hartman & Czyz 2015; George et al. 2016; Hua et al. 2018).

Although the various functions of the Mitf gene have been studied extensively, very little is known about its effect on the development of the vasculature and function in general and even less on the retinal vasculature, although retinal structure and function has been examined in some mice with pathogenic variations (Garcia‐Llorca et al. 2019). The MITF‐Tfe subfamily of transcription factors, including MITF, have been shown to regulate the expression of VEGF in the RPE (Ford & D'Amore 2012). Another member of the MITF‐Tfe subfamily, Tfeb, has been shown to regulate the expression of *Vegf* and possibly other genes of importance for normal vascularization of the placenta (Steingrimsson et al. 1998). The discovery of mouse models of vascular features in the retina that resemble those in human diseases are of great importance as such models may be of use in developing and testing potential treatments of these diseases. The goal of the present study was to analyse the retinal vasculature in mice carrying pathogenic variants in the Mitf gene, which in the eye is solely expressed in the RPE. For the purpose of analysing retinal vessel diameter in mice, we developed software that determines the diameter of fluorescence filled retinal vessels.

## Methods

### Animals

Both wild type mice (C57BL/6J) and mice carrying pathogenic variants in the Mitf gene were examined in this study. All mice examined were females, 22–25 grams in weight, and had free access to food and drinking water during maintenance. All experiments were approved by the Icelandic Food and Veterinary Authority (MAST licence No. 2017‐04‐03). Animals were kept in a 12‐hour light : 12‐hour dark cycle. The mice were 3 months of age at the time the experiments were carried out. Mice were anaesthetized by an intraperitoneal injection of 40 mg/kg ketamine and 4 mg/kg xylazine prior to imaging. Corneal anaesthesia and mydriasis were produced using tetracaine (1% MINIMS, Bausch&Lomb) and tropicamide (10 mg/ml Mydriacyl, Alcon Laboratories) respectively. Methylcellulose (2% Methocel, OmniVision) along with contact lenses were applied to the cornea afterwards, to ensure that the cornea remained moist and to prevent formation of cataract (Ridder 3rd et al. 2002). All experimental procedures were carried out in compliance with the Association for Research in Vision and Ophthalmology Statement for the Use of Animals in Ophthalmic and Visual Research.

### Mice carrying *Mitf* pathogenic variants examined

The pathogenic variations examined for the vessel diameter measurements are *Mitf* ^mi‐vga9^^/+^ (heterozygotes) (Hodgkinson et al. 1993) and *Mitf* ^mi‐enu22(398)^ */Mitf* ^mi‐enu22(398)^ (homozygotes) (Bauer et al. 2009). These mice carrying pathogenic variants have been shown to have normal retinal function and structure, but a hypopigmented fundus (Garcia‐Llorca et al. 2019). A total of 6 mice were examined in each group, including wild type mice. Only the left or right eye, determined randomly, of each mouse was photographed. We found previously that the RPE is thinner in *Mitf* ^mi‐enu22(398)^ */Mitf* ^mi‐enu22(398)^ mice than in *Mitf* ^mi‐vga9/+^ mice; both have normal vision as determined by electroretinography (ERG) measurements (Garcia‐Llorca et al. 2019). The RPE in *Mitf* ^Mi‐Wh/+^ mice are profoundly degenerated and is entirely missing from *Mitf* ^Mi‐Wh/mi^ */Mitf* ^mi^ mice. ERG recordings performed in the same study showed that the *Mitf* ^Mi‐Wh/+^ and *Mitf* ^Mi‐Wh/mi^ */Mitf* ^mi^ mice had completely flat responses to all stimuli. Both genotypes are thus blind, having severe retinal degeneration and lacking both the photoreceptor and outer plexiform layers (Garcia‐Llorca et al. 2019; Table 1).

**Table 1:** The *Mitf* pathogenic variants examined and their characteristics. Source: (Arnheiter 2010; Lu et al. 2010).

Symbol	Mode of induction	Heterozygote	Homozygote	DNA Lesion	Effects on the protein level
*Mitf* ^mi‐vga9^	Transgene insertion	Normal	White coat, eyes red and small; inner ear defects	Transgene insertion and 882‐bp deletion	Reduction in *Mitf* expression (Hodgkinson et al. 1993)
*Mitf* ^mi‐enu22(398)^	Ethylnitroso‐urea	Normal	Significant portion of coat normally pigmented, extensive white spots on belly and over the rest of the body, normal eye size	Stop codon in exon 2A	The lesion terminates the MITF protein prematurely, resulting in a short peptide (Bauer et al. 2009)
*Mitf* ^Mi‐Wh^	Spontaneous or X‐irradiation	Coat colour lighter than dilute(d/d); eyes dark ruby; spots on feet, tail and belly; inner ear defects	White coat; eyes small and slightly pigmented; spinal ganglia, adrenal medulla, and dermis smaller than normal; inner ear defects; reduced fertility	I212N	Unknown
*Mitf* ^mi^	X‐irradiation	Iris pigment less than in wild type; spots on belly, head and tail	White coat, eyes small and red; deficiency of mast cells, basophils, and natural killer cells; spinal ganglia, adrenal medulla, and dermis smaller than normal; incisors fail to erupt, osteopetrosis; inner ear defects	3‐bp deletion in basic domain	Unknown

### Fluorescein angiography

Fluorescence sodium salt 98.5–100.5% (100 mg/ml) diluted with distilled water (final concentration 50 mg/ml) was administered in one bolus (150 μl) by intraperitoneal injection. Fundus images were obtained with a Phoenix Micron IV fundus camera system (Phoenix Technology Group, Pleasanton, CA, USA), with a standard set of excitation and barrier filters for fluorescein angiography.

### Pre‐processing and analysis of the retinal vasculature

**Fig. 1:**
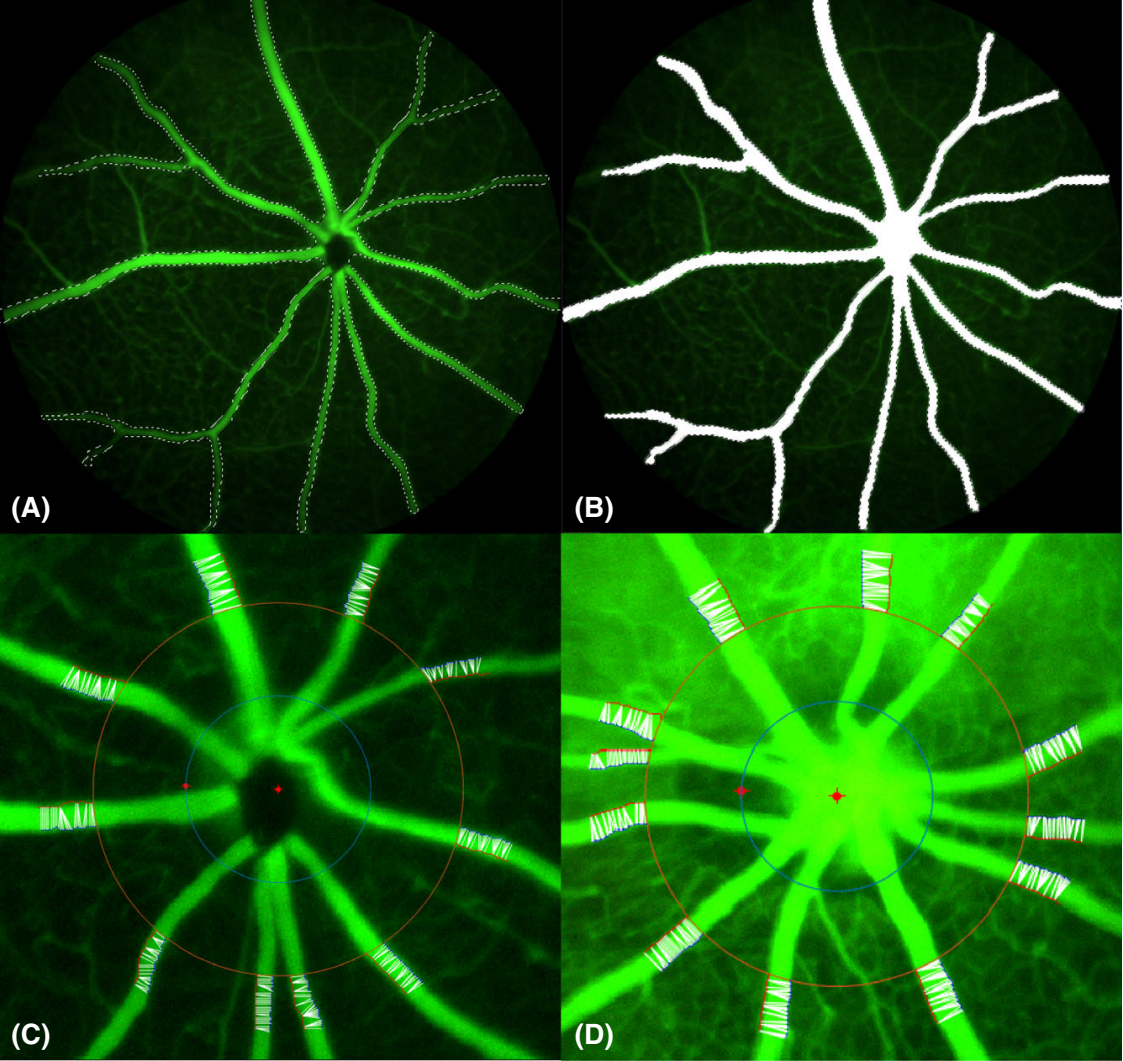
Processing of fundus images prior to analysis and detection of vessel diameter. (A) Fundus image from a wild type mouse, with selection of vessels indicated by white bars. (B) fundus image where vessels have been filled with a high‐intensity white background. (C) Fundus image from a wild type mouse, with a red inner circle 2 times the radius of the optic disc superimposed. The radius of an outer circle is twice that of the inner circle. The white lines across the vessels represent measurements of the shortest distance between points. (D) Fundus image from a *Mitf* ^mi‐enu22(398)/^ *Mitf* ^mi‐enu22(398)^ mouse. Markings of inner and outer circles around the optic disk, and lines on vessels as in (C).

A MATLAB 8.0 program (The MathWorks, Inc., Natick, MA, USA) was created to measure the diameter of mouse retinal vessels. The program measures the intensity of pixels in mouse fundus images in a circular, clockwise direction from the centre of the optic disc at a radius that is twice that of the optic disc. This was done to avoid any error in measurements caused by different contrast and colour of the optic disk as compared to the rest of the fundus. The pixel intensity of retinal vessels in the fundus images from mice carrying the pathogenic variation *Mitf* ^mi‐enu22(398)^/*Mitf* ^mi‐enu22(398)^ was found to be close to the intensity of the remainder of the fundus, and thus with a reduced likelihood to reach the threshold intensity set for vessels. Thus, the images needed to be pre‐processed before they were analysed by the software, the selection tool in Adobe Photoshop CC 2017 (Adobe Inc., San Jose, CA, USA) was used to select the vessels (Fig. 1A) in the images, and they were subsequently removed from the image and replaced by a maximum‐intensity white background (RedGreenBlue settings at = 255, 255, 255) (Fig. 1B) thus enhancing the contrast between the vessels and the rest of the fundus. Pre‐processing of the images was done by one person, and analysis of the retinal vasculature was done by another investigator who as unaware of the genotype. For the purpose of this study, it was not necessary to select the whole vessel bed as shown in Fig. 1B, only that part of it which was within the measurement range. A subroutine was added to the software that allowed for reading both the original image and the processed image concurrently. Although the program added points to the processed image, they were displayed to the user as if they were on the original image. The user thus determines if the measurements are accurate, and if not, can make changes to the selection. The software intensity threshold was set to 80% on all the images analysed. After the vessels had been carefully selected, they were run through the MATLAB program. The validation of the MATLAB program consisted of assessing the repeatability of the results. Several images from the same eyes were obtained consecutively and analysed with the software. Based on the program output the selections were improved if a point did not fall exactly on the margin of a vessel. This was done the same way for all the images. When pixels above and below a user specified threshold are encountered the software marks those pixels with a starting point or end point, respectively (Fig. 1C, showing an image from a wild type mouse). This procedure is repeated 30 times in the analysis of the image, each time moving further in small steps from the optic disc centre. The program then goes through each of the start points and finds the shortest distance to one of its 30 corresponding end points. The result is an image containing the points and a white line drawn between them that represents the measurements; examples of such images from a wild type mouse and *Mitf* ^mi‐enu22(398)^ */Mitf* ^mi‐enu22(398)^ mouse carrying pathogenic variant are shown in Fig. 1C, and Fig. 1D. The software then exports the measurement values for each vessel into an Excel document which provides a calculation of the mean, median and standard deviation of these values.

### Identification of venules and arterioles in fundus images

An alternating vascular pattern was assumed when determining which vessels were arteries or veins, as has been shown previously in about 80% of eyes in C57BL/6J mice (McLenachan et al. 2015; Rust et al. 2019). This alternating pattern has previously been confirmed in wild type mice by dynamic fluorescent angiography experiments, in which a fluorescent salt solution is injected into the femoral vein and a video of fundus images is acquired, showing filling of the retinal vasculature, and a clear time difference between arteries and veins (Greferath et al. 2014). In addition, the shape of the main types of vessels appears different in fundus images, where the veins tend to be wider, with fewer curves, and which appear to follow a more direct path than the arteries. In addition, in C57BL/6J (wild type) mice it has been found that in fundus angiography photographs of the kind used in the present study, the major retinal arterioles and venules are present on the surface of the retina at different focus levels, with the arterioles at a more superficial level of focus, while the venules are at deeper levels, which allows for identifying fundus angiography images of the kind used in this study (McLenachan et al. 2015). These patterns fit with a detailed three‐dimensional modelling of the mouse retinal vasculature based on vascular staining, high throughput microscopy and image analysis (Hua et al. 2018; Rust et al. 2019).

### Statistical analysis

Collation of data and all calculations were performed in Excel (Microsoft Corp, Redmond, WA, USA), while bar chart graphs were made in SigmaPlot 13.0 (Systat Software Inc, San Jose, CA, USA). Statistical analysis was done in SigmaPlot 13.0. A Student's two‐tailed *t*‐test was performed to compare the means between groups, and any differences with p‐values below 0.05 were considered statistically significant. However, in instances where the test for normality failed, the non‐parametric Mann–Whitney Rank Sum Test was applied. When calculating the mean vessel diameter, the mean diameter for arteries, veins and both were calculated using the mean diameter of each vessel (a mean of the 29 measured values extracted with the MATLAB program). All subsequent data analysis was done using this mean (*i.e*. one value for the arterial, venular and total diameter respectively for each image analysed).

## Results

**Fig. 2:**
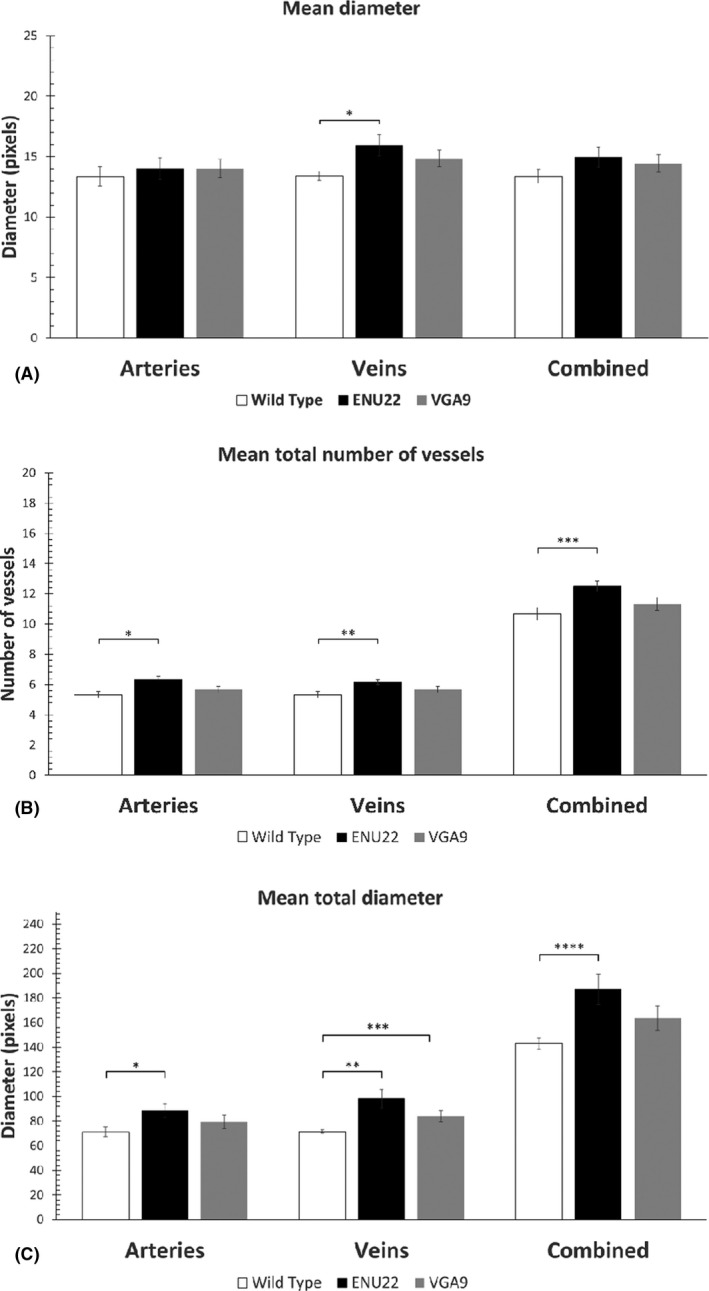
(A) The diameter (in pixels) of arteries, veins and both in the three genotypes examined. * p = 0.026. (B) Mean number (in pixels) of arteries, veins and both in the three genotypes. 18.8% *p = 0.026. **p = 0.041. ***p = 0.026. (C) Mean combined diameter (mean number of vessels x mean diameter, in pixels) for the three genotypes. *p = 0.024. **p = 0.004. ***p = 0.030. ****p = 0.007. ENU22 = *Mitf* ^mi‐enu22(398)/mi‐enu22(398)^, VGA9 = *Mitf* ^mi‐vga9/+^. Error bars denote one standard error around the mean. *N* = 6 in all groups.

The method used to analyse the retinal vasculature is depicted in Fig. 1 and was applied to mouse fundus images from all the mice examined. It involved measuring the pixel intensity in mouse fundus images within a fixed radius from the optic disk, and to detect the retinal vessels in that area based on intensity with a software for that purpose written by the authors. The results are presented in Fig. 2 and Table 2. Figure 2A shows the mean diameter of arteries and veins separately in the retinal vasculature of wild type mice and mice with the *Mitf* ^mi‐enu22(398)*/*^ *Mitf* ^mi‐enu22(398)^ and *Mitf* ^mi‐vga9/+^ pathogenic variations, and the combined diameter of both arteries and veins in these animals, to account for any differences in the number of vessels in the retinas of the mice, which may affect the mean diameter.

**Table 2:** Comparison between mean diameter, mean combined diameter and mean number of vessels of the retinal vasculature in all genotypes examined.

Mean diameter	Diameter (pixels)	% Increase	p‐value
Arteries			
Wild Type	13,3684		
*Mitf* ^mi‐enu22(398)/mi‐enu22(398)^	14,0229	105	0.598
*Mitf* ^mi‐vga9/+^	14,0109	105	0.579
Veins			
Wild Type	13,4198		
*Mitf* ^mi‐enu22(398)/mi‐enu22(398)^	15,9425	119	0.026*
*Mitf* ^mi‐vga9/+^	14,8438	111	0.101
Combined			
Wild Type	13,3941		
*Mitf* ^mi‐enu22(398)/mi‐enu22(398)^	14,9683	112	0.152
*Mitf* ^mi‐vga9/+^	14,4273	108	0.288

Mean number of vessels	No. vessels	% Increase	p‐value
Arteries			
Wild Type	5,3333		
*Mitf* ^mi‐enu22(398)/mi‐enu22(398)^	6,3333	119	0.026*
*Mitf* ^mi‐vga9/+^	5,6667	106	0.290
Veins			
Wild Type	5,3333		
*Mitf* ^mi‐enu22(398)/mi‐enu22(398)^	6,1667	116	0.041*
*Mitf* ^mi‐vga9/+^	5,6667	106	0.290
Combined			
Wild Type	10,6667		
*Mitf* ^mi‐enu22(398)/mi‐enu22(398)^	12,5000	117	0.026*
*Mitf* ^mi‐vga9/+^	11,3333	106	0.290

Mean combined diameter	Diameter (pixels)	% Increase	p‐value
Arteries			
Wild Type	70,9570		
*Mitf* ^mi‐enu22(398)/mi‐enu22(398)^	88,5775	125	0.024*
*Mitf* ^mi‐vga9/+^	79,3658	112	0.236
Veins			
Wild Type	71,2680		
*Mitf* ^mi‐enu22(398)/mi‐enu22(398)^	98,7619	138	0.004**
*Mitf* ^mi‐vga9/+^	83,9785	118	0.030*
Combined			
Wild Type	142,8704	100	
*Mitf* ^mi‐enu22(398)/mi‐enu22(398)^	187,1037	131	0.007**
*Mitf* ^mi‐vga9/+^	163,5099	114	0.093

Note: **Mann–Whitney Rank Sum Test applied, p <0.05. *Two‐tailed *t*‐test, p <0.05.

All significant p‐values in the table are highlighted by bold.

Figure 2(A) indicates that the mean diameter of the retinal arteries in wild type, *Mitf* ^mi‐enu22(398)*/*^ *Mitf* ^mi‐enu22(398)^ and *Mitf* ^mi‐vga9/+^ mice carrying pathogenic variants was not significantly different statistically (Fig. 2A, left; Table 2). However, the mean diameter of the retinal veins in mice with the *Mitf* ^mi‐enu22(398)*/*^ *Mitf* ^mi‐enu22(398)^ pathogenic variation was 18.8% larger than in wild type mice (p = 0.026) (Fig. 2A, middle). When the mean diameter of both arteries and veins for each genotype was combined, the difference between the diameter of retinal vessels in the *Mitf* carrying pathogenic variants as compared to wild type mice did not reach statistical significance (Fig. 2A, Table 2). The mean total number of retinal arteries and veins was determined from fundus photographs of one eye from six mice of each genotype, and the results are presented as means ± SEM in Fig. 2B and Table 2. Figure 2B indicates that the *Mitf* ^mi‐enu22(398)*/*^ *Mitf* ^mi‐enu22(398)^ mice have 17.2% more retinal arteries (p = 0.026; Fig. 2B, left) and 15.6% more retinal veins (p = 0.041; Fig. 2B, middle) than wild type mice. When the mean number of both retinal arteries and veins for the *Mitf* ^mi‐enu22(398)*/*^ *Mitf* ^mi‐enu22(398)^ mice were combined, it was found to be 17.2% higher than in wild type mice (p = 0.026; Fig. 2B, right). The number of retinal arteries and veins in *Mitf* ^mi‐vga9/+^ mice were not significantly different from those of wild type or *Mitf* ^mi‐enu22(398)*/*^ *Mitf* ^mi‐enu22(398)^ mice (Fig. 2B, Table 2). In some cases, there was a difference in the number of vessels and to take this into account, we also compared the mean total vessel diameter of each retinal vessel type by multiplying the mean diameter of the respective vessel type with the number of vessels. This value indicates the extent of vascularization of each vessel type in each genotype. There was a significant 24.8% increase in mean combined arterial diameter in mice with the *Mitf* ^mi‐enu22(398)*/*^ *Mitf* ^mi‐enu22(398)^ pathogenic variants compared to wild type (p = 0.024, Fig. 2C, right). A significant 38.6% increase in mean combined venular diameter was observed in *Mitf* ^mi‐enu22(398)*/*^ *Mitf* ^mi‐enu22(398)^ mice as compared to wild type (p = 0.004) (Fig. 2C, middle). A statistically significant 17.8% increase in the mean combined retinal venular diameter was seen in the *Mitf* ^mi‐vga9/+^ mice compared to the wild type mice (Fig. 2C, middle, and Table 2; p = 0.03). When combining the mean total vessel diameter of both retinal arteries and venules, it was found that there was a significant 31% increase in mean combined vessel diameter (Fig. 2C, right; p = 0.007) in *Mitf* ^mi‐enu22(398)*/*^ *Mitf* ^mi‐enu22(398)^ mice as compared to wild type mice, while the mean combined vessel diameter in *Mitf* ^mi‐vga9/+^ mice was not significantly different from wild type.

## Discussion

The present study is the first analysis of the retinal vasculature and retinal vessel diameter in *Mitf* mice carrying pathogenic variants. The main finding of this study is that there is an increased vascularisation in the retinas of the two variant alleles examined, *Mitf* ^mi‐vga9/+^ and *Mitf* ^mi‐enu22(398)*/*^ *Mitf* ^mi‐enu22(398)^. Several possible mechanisms may account for these differences, including increased formation of reactive oxygen species (ROS) in mice that lack *Mitf* (Hua et al. 2018) and changes in VEGF expression in the RPE (Ford & D'Amore 2012), which may lead to alterations in the choroidal vasculature (Saint‐Geniez et al. 2009). Another possibility involves alterations in RPE function or integrity in *Mitf* mice carrying pathogenic variants which in turn may affect RPE dependent mechanisms involved in vascularization or control of retinal vascular function (Garcia‐Llorca et al. 2019).

The findings of the present study suggest that changes in RPE integrity can have profound effects on the retinal vasculature. A greater difference in the vascular diameter compared to wild type in the *Mitf* ^mi‐enu22(398)*/*^ *Mitf* ^mi‐enu22(398)^ mice than the *Mitf* ^mi‐vga9/+^ mice was found, and the RPE is more depigmented in *Mitf* ^mi‐enu22(398)*/*^ *Mitf* ^mi‐enu22(398)^ than *Mitf* ^mi‐vga9/+^ mice carrying pathogenic variants, while choroidal melanocytes appeared to be near absent from the former genotype but present in the latter (Garcia‐Llorca et al. 2019). The RPE cells however, release several pro‐angiogenic and neurotrophic factors, such as VEGF, PEDF, FGF, and PDGF, some of which *Mitf* has been shown to regulate the expression of (Ma et al. 2012). One of these factors, PEDF, has been shown to partially rescue degenerating photoreceptors in mice with the null allele *Mitf* ^mi‐vga9^, indicating that the RPE‐derived trophic factor, and thus MITF, are involved in retinal homeostasis (Chen et al. 2019). The *Mitf* ^mi‐vga9^ null mice show a significantly higher levels of reactive oxygen species (ROS) in both the retina and the RPE, suggesting that *Mitf* dysfunction may lead to oxidative damage in the RPE and the retina (Hua et al. 2018).

The mean venular diameter in mice with the *Mitf* ^mi‐enu22(398)*/*^ *Mitf* ^mi‐enu22(398)^ pathogenic variation was found to be significantly greater than in wild type mice. This indicates that changes in oxygen metabolism have occurred in the posterior chamber of the eyes of these mice carrying pathogenic variations, not due to alterations in MITF but of otherwise unknown mechanisms, which presumably requires moving more blood from the eye than in wild type mice. Since it was found that there was a significant increase in mean total arterial diameter in the mice carrying pathogenic variations, although the increased diameter of individual retinal arteries did not reach significance, it is likely that there is an increased rate of blood flow through the retinal arteries. If there is more afferent blood flow, there also must be a corresponding increase in efferent blood flow. The same applies for the significant 31% increase in the mean total retinal vessel diameter in the *Mitf* ^mi‐enu22(398)*/*^ *Mitf* ^mi‐enu22(398)^ mice that was found, as compared to wild type. However, we do not yet have any measures of retinal vascular blood flow or vessel oxygen saturation from mice carrying *Mitf* pathogenic variants, to test this assumption. No significant difference in the mean retinal venular diameter was found between *Mitf* ^mi‐vga9/+^ mice and wild type mice, while the mean total venular diameter was significantly greater in mice carrying the *Mitf* pathogenic variations. The mice with the *Mitf* ^mi‐enu22(398)*/*^ *Mitf* ^mi‐enu22(398)^ genotype were found to have a significantly higher mean number of arteries, veins and both combined compared to wild type mice, although not all of them showed that phenotype. We found an increase in the mean combined arterial diameter in mice with the *Mitf* ^mi‐enu22(398)*/*^ *Mitf* ^mi‐enu22(398)^ pathogenic variation compared to wild type. This indicates that while each artery was similar in size in both genotypes, the total retinal arterial vascularization in the mice carrying *Mitf* pathogenic variants was more extensive than in the wild type mice. It might be assumed from this that there is an increased arterial blood supply to the retinas of the *Mitf* ^mi‐enu22(398)*/*^ *Mitf* ^mi‐enu22(398)^ genotype. This was an unexpected result and suggests that there may be a change in metabolism or oxygen demand of the retina in these pathogenic variations. Furthermore, it suggests that the retinal arterial development is normal in this pathogenic variation. However, these findings need to be considered with some caution, since it has been found in C57BL/6J mice that about 15% of adult mice have the hyaloid artery remaining, and that 80% have an alternating pattern of arteries and veins in the fundus, with the rest having a non‐pairing additional arteriole (McLenachan et al. 2015). We did not see clear evidence of the hyaloid artery remaining in any of our mice, regardless of genotype, and none of our C57BL/6J mice had an additional non‐pairing arteriole, but the mice carrying pathogenic variants, in particular the *Mitf* ^mi‐enu22(398)*/*^ *Mitf* ^mi‐enu22(398)^ genotype, had a significantly higher mean number of vessels, both arteries and veins, as a group, indicating that there is a trend towards a more extensive retinal vascularization in these genotypes.

The study has several limitations, one of which is that because only fundus imaging was used for obtaining images of the retinal vasculature, we can only draw conclusions about the superficial layers of that vasculature in our genotypes, while any changes in the deeper layers are still unknown. The *Mitf* ^mi‐enu22(398)*/*^ *Mitf* ^mi‐enu22(398)^ mice had an increase in background intensity in the fundus images. This may have been caused by leakage of the vessels which may in turn affect vascular diameter, or it may be due to hypopigmentation of the fundus, with the latter being more likely (Garcia‐Llorca et al. 2019). It is also possible that the background in the fundi of some of the *Mitf* ^mi‐enu22(398)*/*^ *Mitf* ^mi‐enu22(398)^ mice reach a higher level than the 80% threshold, but this would have been detected by the software and is thus also likely to be a negligible error. In two animals, one C57BL/6J mouse and one *Mitf* ^mi‐vga9/+^ mouse, the vessels branched within the range that the program was measuring (an example of this is shown in Fig. 1D). In those cases, the diameter of the circle in which the program measures vessel diameter was adjusted. The adjustment made was minimal in these cases so that it started to measure just barely past the branch‐point. Nevertheless, branching is a factor that influences the measurements and needs to be carefully considered while performing the analysis with the software. For future development it would be desirable to have the process of determining vascular diameter completely automatic, but which so far has proved to be a difficult task due to the differences in fluorescence intensities, potential vasculature leakage and other differences in the retinal vasculature between mice carrying pathogenic variants.

## Conclusions

We found a statistically significant difference between the retinal vasculature in wild type mice and mice carrying pathogenic variants in the *Mitf* gene, *Mitf* ^mi‐enu22(398)^ */Mitf* ^mi‐enu22(398)^ and *Mitf* ^mi‐vga9/+^. The difference is more profound between *Mitf* ^mi‐enu22(398)*/*^ *Mitf* ^mi‐enu22(398)^ and wild type mice, showing an increase in arterial and venular retinal vasculature. The difference is less between the mice with the *Mitf* ^mi‐vga9/+^ genotype and wild type mice, in which there is an increase in venular, but not arterial, retinal vasculature diameter. This indicates that there is an increase in the retinal vasculature in both these pathogenic variations, possibly due to an increase in metabolic needs, which needs to be investigated further. Changes in the choriocapillaris in these mice have not yet been examined but might shed further light on the effects of *Mitf* on vascular structure and function at the back of the eye.
